# ”Putting words to their feelings”– civic communicators’ perceptions and experiences of an in-depth course on mental health for newly settled refugee migrants in Sweden

**DOI:** 10.1186/s12913-023-09524-2

**Published:** 2023-05-19

**Authors:** Maissa Al-Adhami, Josefin Wångdahl, Raziye Salari, Eva Åkerman

**Affiliations:** 1 Department of Women’s and Children’s Health, Research and Learning for Sustainable Development and Global Health (SWEDESD), Uppsala University, Box 564, Uppsala, 751 22 Sweden; 2 Department of Public Health and Caring Sciences, Child Health and Parenting (CHAP), Uppsala University, Box 564, Uppsala, 751 22 Sweden; 3 Department of Neurobiology, Care Sciences and Society, Aging Research Center, Karolinska Institutet & Stockholm University, Tomtebodavägen 18A, 171 77 Solna, Sweden; 4 Department of Women’s and Children’s Health, Karolinska Institutet, Tomtebodavägen 18A, 171 77 Stockholm, Sweden

**Keywords:** Mental health training, Refugee migrants, Mental health literacy, Health promotion, Sweden

## Abstract

**Background:**

Newly settled refugee migrants face psychological stressors stemming from pre-, during- and post-migration experiences. In Sweden, mental health promotion is part of the health module in the civic orientation classes for newly settled refugee migrants. Training courses are offered to civic communicators and workshop leaders to facilitate communication about mental health; however, the training is seldom evaluated. In the current study, we aim to explore civic communicators’ perceptions and experiences of an in-depth mental health training course in relation to observed needs among newly settled refugee migrants.

**Method:**

We interviewed ten civic communicators that had partaken in the in-depth training course on mental health. All respondents had prior migratory experience and worked as civic communicators in their native languages. The interviews were semi-structured and data were analyzed using thematic analysis.

**Results:**

Three themes were identified: (1) Intertwined mental health needs related to migration, (2) Multi-layered barriers to addressing mental health, and (3) Becoming aware of the mental health journey. One overarching theme was arrived at through synthesizing the three themes ‘Acquired new tools to lead reflective conversations about mental health and well-being’.

**Conclusion:**

The in-depth mental health training course led to the attainment of new knowledge and new tools enabling civic communicators to lead reflective conversations about mental health and well-being with newly settled refugee migrants. Mental health needs were related to pre- and post-migration experiences. Barriers to talking about mental health included stigma and a lack of arenas to promote the mental health of refugee migrants. Increasing knowledge among civic communicators can facilitate the promotion of mental self-help capacity and resilience among newly settled refugee migrants.

**Supplementary Information:**

The online version contains supplementary material available at 10.1186/s12913-023-09524-2.

## Background

In the last decade, Sweden has received a relatively large number of refugees compared to other European countries, fleeing the war in Syria and other conflicts. From 2015 to 2019, over 250 000 permanent or temporary residence permits were granted based on refugee status, subsidiary protection status and family reunification [1]. Recently, in the first half of 2022, about 40 000 Ukrainian refugees received temporary residence permits [1].

The mental health of newly settled refugee migrants is affected by structural and contextual barriers in the resettlement phase, such as inadequate housing and employment opportunities [2, 3], acculturation stress, isolation and discrimination [4–6]. The need for mental health promotion aimed at refugee migrants has been increasingly recognized by regional European and local authorities, leading to the initiation of public and non-profit services and programs [7–10] but we need a better understanding of how mental health programs targeted at newly settled refugee migrants should be designed and delivered [11, 12]. To reduce the impact of mental ill health, it is essential to ensure that clinical, as well as the non-clinical staff who lead interventions and programs aimed at refugee migrants, possess relevant knowledge and skills [13, 14].

Despite being heterogeneous in many aspects, at group level, refugees are more likely to suffer from mental ill health and experience specific disorders such as post-traumatic stress and depression than the general populations in hosting high-income countries [15–19]. The WHO defines mental health as “a state of well-being in which an individual realizes his or her abilities, can cope with the normal stresses of life, can work productively and is able to make a contribution to his or her community” [20]. In the case of refugees, mental health is affected by factors occurring pre-, during- and post-migration. Examples of pre-migration adverse factors are persecution, physical and psychological violence, forced migration and involuntary separation from family members [2, 21]. During the flight, hazardous elements include traveling in unsafe ways, witnessing death and violence, and being detained at borders [22]. In the early post-migration phase, mental stressors are linked to, for example, uncertainty about the asylum process and worrying about family members left behind [23] as well as structural and contextual factors linked to the resettlement [3, 24]. Refugee migrants are also more likely to have difficulties accessing and utilizing mental health services in comparison with the majority population [25, 26], because of language barriers, poor knowledge about mental health as well as the health care system in hosting countries [26–28]. Other reasons for not seeking mental health care include fear of disclosure due to the stigma surrounding mental ill-health common in many cultures as well as different help-seeking behaviors and coping mechanisms [29–31]. For example, Syrian migrants report that they turn to their own close family and friends for support when facing depression [32]. However, with migration, social networks are interrupted and it takes time to form new ones [33, 34].

Mental health promotion for refugee migrants has increasingly focused on individuals' own strengths, abilities and resilience [11]. For milder mental illness, interventions that promote knowledge and utilization of various self-help strategies may be sufficient to prevent the development of clinical mental illness [13]. At the individual level, promoting mental health for newly settled migrants is important not only to promote resilience against post-migration stressors but also to increase the likelihood of a successful establishment and resettlement. Good mental health is, for example, associated with better language learning outcomes [35, 36], which increases an individual's ability to find employment which in turn is an important factor for long-term mental health and well-being [37].

In Sweden, newly settled refugee migrants with a residence permit partake in a Civic Orientation course (CO) that is part of a two-year Introduction Program (also referred to as the Establishment Program).The program was implemented nationally in 2010, replacing and streamlining earlier Introduction programs with varying content and delivery, provided at the municipality level. The aim of the Introduction Program is to facilitate social integration and access to work through language courses, CO and work counselling activities (Law 2010:197). The CO includes a minimum of 100 h of information about society (e.g., how society is organized, everyday life, rights and obligation) as well as health information (e.g., the right to health care, how to take care of one’s health in Sweden). The provision of CO is the responsibility of the municipalities that cooperate and coordinate regionally. Health needs and health promotion activities for newly settled migrants in the establishment phase have been evaluated in recent years [38–41]. There is, however, limited knowledge about training programs directed toward non-clinicians to prepare them for working with mental health promotion, and how these programs are perceived by non-clinicians vis-à-vis observed needs in refugee groups [12, 42]. This knowledge is needed to understand in what ways these training courses and programs are beneficial, and how they may be optimized to contribute to the alleviation of mental ill health among refugee migrants.

### Training programs and in-depth training in mental health for civic orientation communicators

**Fig. 1:**
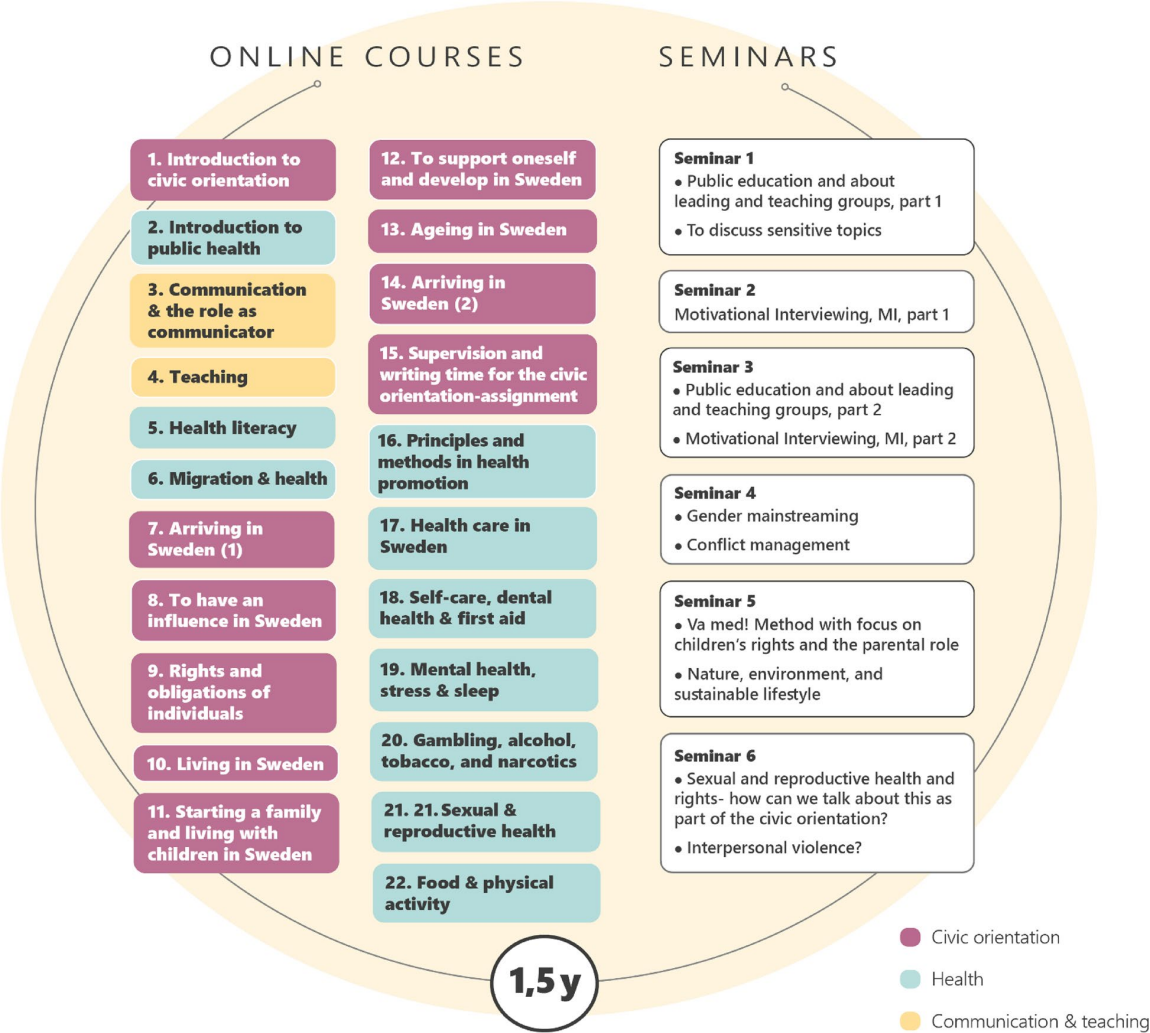
Milsa education program for civic- and health communication

CO is provided in the major native languages of newly settled refugee migrants and led by teachers referred to as *civic communicators*. The majority of civic communicators have experienced migration as refugees themselves, have language, culture competence from their countries of birth, and are familiar with the language, society and culture of Sweden. In response to the needs identified by stakeholders to professionalize the role of civic communicators and provide equal civic and health orientation nationwide, an EU-funded national program called “*Education platform for civic- and health communication”* was developed and implemented in 2017–2021 [43]. The program was part of MILSA (Support Platform for Migration and Health), a collaborative national platform for public stakeholders and universities on migration and health. The program included CO themes and topics such as health sciences, health promotion, pedagogy and communication (Fig. 1). Two hundred civic communicators from all parts of Sweden participated in the program, which was delivered through a combination of online and face-to-face teachings.

An additional in-depth training course on mental health was offered to smaller groups of those who had taken part in the MILSA training program (ten at a time). The objectives of the in-depth training course were two-fold; to provide knowledge and understanding of mental health and its promotion in relation to migration and being new in Sweden and to equip civic communicators to lead conversation circles aimed at refugee migrants who experience mental ill health in the early post-migration phase. A conversation circle in this setting is a group activity where participants meet regularly (in small groups) during a period to learn more about a specific subject through communication and conversations led by a trained leader.

In study, we aim to explore civic communicators’ perceptions and experiences of the in-depth training course on mental health in relation to observed psychological needs among newly settled refugee migrants.

### Theoretical framework

Mental health literacy (MHL) will be used as a framework to discuss the findings of the study. MHL is a construct derived from the concept of health literacy, which can be defined as the abilities and resources needed to obtain and maintain health through finding, understanding, assessing, and applying health information [44]. MHL can be defined as: “Understanding how to obtain and maintain positive mental health; understanding mental disorders and their treatments; decreasing stigma related to mental disorders; and, enhancing help-seeking efficacy, i.e., knowing when and where to seek help and developing competencies designed to improve one’s mental health care and self-management capabilities” [45, 46]. Health literacy should not be regarded as an individual characteristic; rather it is “co-created” with various societal organizations such as health care services and schools [47]. As in the case of health literacy, MHL needs to be context specific, i.e. tailored, developed and applied in everyday life situations and integrated into existing structures, such as schools and community organizations [45]. In health promotion activities targeted at newly settled migrants, increased MHL is assumed to positively affect mental health and well-being through different pathways such as attitudes, health behaviors and health-seeking behavior [12].

## Methods

### Study design

We conducted an explorative qualitative study, based on individual interviews with civic communicators. Individual interviews are considered appropriate for exploring individuals’ perceptions and experiences, specifically suitable for exploring topics of a potentially sensitive nature [48].

### Setting

This study was part of a larger evaluation of MILSA's in-depth training course on mental health and well-being. The in-depth mental health training course took place at a conference center in Stockholm, and was given three days in a row, on two occasions at approximately 6-month intervals; in spring 2020 and autumn 2020 respectively. Furthermore, a three-hour ‘refresher’ opportunity was offered online approximately one month before the second on-site training occasion. The training was led by a team of professionals with specialist competence in the course’s themes. MILSA's circle leader guide *Ways forward after the flight* was used in the training. The training was experience- and dialogue-based, meaning that it combined theory, reflection, discussion and practical exercises.

**Fig. 2:**
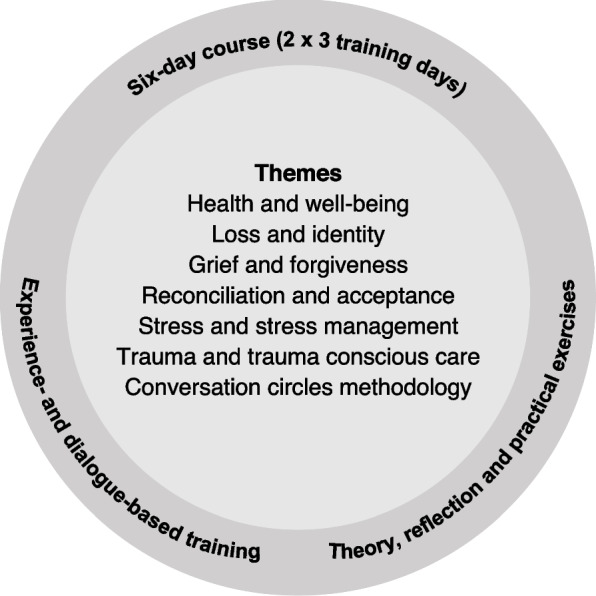
Content and In-depth mental health training course

It included six themes: health and well-being, loss and identity, grief and forgiveness, reconciliation and acceptance, stress and stress management, trauma and trauma-conscious care (Fig. 2). In addition, conversation circle methodology was taught, including pedagogical methods, the role of the circle leader, and film as a tool for conversation. All themes, including the methodology are described in more detail in Supplementary file 1.

### Data collection

We used purposive sampling for recruiting respondents. The inclusion criteria were to have participated in MILSA’s educational program in civic- and health communication and fully completed the in-depth training course on mental health. All 10 that had participated in the in-depth course on mental health at the time of the study, were invited and consented to participate in the study. The interviews were conducted in September and October 2020 (about one month after the last training session). Due to the restrictions enforced by the covid-19 pandemic, all interviews were conducted via the electronic platform Zoom. We developed a semi-structured interview guide with open-ended questions and used probes when needed. The interview guide included questions about thoughts and experiences of the course, learning and reported new knowledge and perceptions about the course related to their role as civic communicators (Supplementary file 2). Each interview lasted for about 60 min and was recorded (visual and audio). The interviews were then transcribed verbatim from audio files by a professional transcriber according to Mc Lellan’s data preparation and transcription guidelines [49]. All interviews were conducted in Swedish by the second author (JW), a female researcher born in Sweden with extensive experience in health literacy research and qualitative studies involving refugee migrants. The interviewer met the respondents as a lecturer in the MILSA educational program but was not involved in the training of the in-depth course.

### Participants

Eight of 10 respondents were women and two were men. The median age was 41 years (Table 1). They were born in seven different countries and had lived in Sweden for nine years on average. All respondents had an academic education and were full-time employees as communicators or coordinators within the CO. They were living in different parts of Sweden and had worked six years on average within the CO. The majority worked with Arabic-speaking groups leading two to four CO groups per semester. When the interviews were conducted, two respondents had already started their first conversation circle on mental health for newly settled migrants and completed five out of the twelve occasions. The other respondents had not yet started one.

**Table 1:** Respondents’ characteristics

**Age**	**Median (range)**
Years	41 (35–54)
**Sex**	**n**
Woman	8
Man	2
**Country of birth**	**n**
Arabic speaking countries	8
Non-Arabic speaking countries	2
**Years living in Sweden**	**Median (range)**
Years	9 (5–30)
**Education level**	**n**
More than 12 years (university or other higher education)	10
**Primary working language in CO**	**n**
Arabic	6
Other languages	4

### Data analysis

**Fig. 3:**
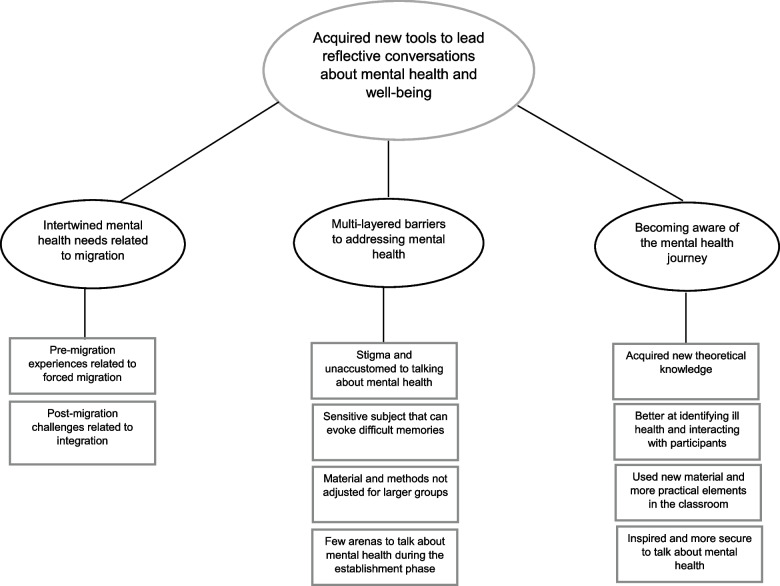
Thematic map

Data were analyzed using inductive thematic analysis described by Braun & Clarke [50]. As a first step, authors (JW, MA, EÅ) familiarized themselves with the data by reading the transcripts. The authors then developed initial codes independently. This was done by identifying interesting descriptive and latent features in the data and assigning codes to them in a systematic way across all transcripts. The codes were inserted directly on the right-hand margin of the transcripts and then compared and defined. All related codes explaining similar patterns or features were collated to form preliminary sub-themes that were. The subthemes were then (1) checked against the codes and refined, and (2) further organized into themes and one overarching theme as illustrated in the thematic map (Fig. 3). To validate the findings, the authors (JW, EÅ, MA) compared and discussed the sub-themes and themes that they had developed independently. MA then reviewed and refined the sub-themes and themes before a final triangulation meeting was held to finalize themes including the overarching theme (illustrated in Fig. 3). Throughout the analysis, there was continuous reviewing of the sub-themes and themes that included going back to the transcripts and codes, in order to meet consensus and to ensure that the themes reflected the data set. Quotations were used to highlight the main findings.

The research team consisted of four female researchers; one PhD-student, two post-docs and one associate professor. Together they have broad experience within the field of migration and health and qualitative research methodology. They have been involved in research and practice involving refugees and migrants within the field of health promotion and health literacy (JW, MA), sexual and reproductive health and rights (EÅ) and programs to promote parents’ and children’s health (RS). One of the four authors is Swedish-born and three have an immigrant background with cross-cultural skills relevant to the study.

### Ethics

Ethical approval was obtained from the Swedish Ethical Review Authority, Uppsala (registration number 2019–00,035). The respondents were informed orally and by email about the study aim and procedure in connection with taking the course. They were informed that participation was voluntary, and that they could withdraw from it at any time without any consequences. The respondents were also informed that their answers would remain confidential, and that all identifying information would be deleted from the transcripts. Similarly, in the case of quotations, neither age, gender nor country of birth would be disclosed so that no link could be made to any of the respondents. All informants gave informed consent before they participated in the interviews. All informants agreed to have the interviews recorded.

## Results

The analytical process led to the crystallization of three themes and one overarching theme*.* The overarching theme – ‘*Acquired new tools to lead reflective conversations about mental health and well-being’ –* was arrived at through synthesizing the three themes; (1) *Intertwined mental health needs related to migration,* (2) *Multi-layered barriers to addressing mental health* and (3) *Becoming aware of the mental health journey*. Each of the themes have subthemes outlined below and in Fig. 3.

### Intertwined mental health needs related to migration

The first theme sums up reflections and discussions around the perceived needs of mental health among newly settled refugee migrants. The needs were seen as intertwined, i.e., related to both pre- and post-migration events and conditions. Commonly, the respondents perceived that there is an extensive need for mental health information among newly settled refugee migrants, even if they described different levels of mental ill health within the groups that they taught. The respondents also stressed the importance of viewing the participants in classes as individuals and not making assumptions based on country of origin or migration status.

#### Pre-migration experiences related to forced migration

This subtheme refers to the respondents’ experiences and perceptions about mental health needs related to the migration journey of newly settled migrants in their classes. The needs that were articulated by the respondents were seen as originating from often unpleasant memories and traumatic experiences that had primarily occurred pre- and during the migration to Sweden. Examples of traumatic events pre- and during migration were fleeing from war (forced migration), leaving loved ones behind, fleeing in unsafe ways and loss of family members during flight. One respondent recalled how a participant in her CO classroom had shared a strong memory of losing her child in the sea overpass to Europe, which affected everyone in the class, reminiscing similar memories of loss. Several respondents mentioned the need to follow up on participants in their classrooms with especially traumatic experiences and refer them to appropriate health care.

The subtheme also resonated with several of the respondents themselves that had difficult memories of traumatic events and difficulties from prior phases of migration, despite having spent many years in Sweden.

*We come from war and work with these people, so it is also our experiences in some ways. We remember and it comes up to the surface but we try to deal with it as best we can. (respondent 2)*

Some respondents pointed out that shared experiences were the key to participants’ willingness to open up and talk about certain experiences; it was easier to talk to someone who would understand what they had been through.

#### Post-migration challenges related to integration

Post-migration challenges and requirements such as learning the language, finding a job and integrating in Sweden were seen as adding to the mental stress among the newly settled group. Some reflected that an initial positive outlook of having reached safety could later change in face of difficulties, increasing the stress level with time. Mental health needs were perceived to be varied among the migrant group; however, several respondents mentioned finding a job as a common source of stress and worry.

*There is little or no chance to find work (in small municipalities) so they don’t have hope for the future and that’s really bad. It’s of course a part of their stress problems, it could be their old life, maybe parents who need help and so on, but at the same time, there is a lot that affects our mental well-being and it is the future. They know that chances to find work, especially in our small municipality are scarce so that affects their mental health. (respondent 3)*

Another area that came up in several interviews was stress related to parenting issues, a subject that was often discussed in the classrooms. Behavioral issues among children and differences in parenting style in Sweden compared to countries of origin were seen as a source of stress. In addition, the parents’ mental health was also seen as affecting their abilities to attend to and interact with their children. Based on these observed needs, many of the respondents reflected that mental health information and promotion were needed both in the form of conversation circles and as part of the civic orientation.

*If your health isn’t good and the person doesn’t understand what the problem is, they can be healthy physically, but it’s something in your head that make you stressed, then you can’t plan, you can’t concentrate and you can’t raise your children. (respondent 3)*

Several respondents had observed that some of their participants were unaware that they were unwell, thinking that their stress was normal. Respondents reflected that unmet mental health needs of newly settled migrants could affect their potential to benefit from the CO and other introduction activities such as language training and job counseling.

*They need help to integrate into society and it is a very good way to integrate into society, by feeling well. (respondent 1)*

### Multi-layered barriers to addressing mental health

The second theme describes different types of barriers to talking about mental health. Barriers were seen as multi-layered, i.e., connected to both the individual and structural level. In general, the barriers to talking about mental health were discussed by respondents in relation to applying the material and methods in larger classrooms, i.e., civic orientation classes, rather than the conversation circles.

#### Stigma and unaccustomed to talking about mental health

A majority of the respondents reflected on the difficulties to talk about mental health and ill health in larger groups of newly settled migrants. Stigma, shame and fear associated with mental ill health were perceived to be a barrier. However, few respondents used the word ‘stigma’, instead describing that participants in their classrooms were unaccustomed to talking about mental ill health for cultural reasons. Furthermore, some respondents explained that participants in their classrooms often lacked the vocabulary to talk about mental health and instead used words such as ‘being tired’ or just ‘stressed’ for all types of situations and conditions.

*I come from a country where we don’t put much focus on mental health. We are not used to talking about it even if it exists and is brought up. It’s rare that a person talks about it and if they do, and say that they aren’t feeling good, the answer would be: take an aspirin and drink water. (respondent 1)*

A few respondents reported that some of the participants in the civic orientation classes tried to hide or pretend to be feeling well or hide symptoms in fear of it affecting their families and children negatively.

*As I told you, there is fear in some participants, they don’t dare to say that they are not feeling well, or feeling mentally unwell. There is a big fear in them because they think that it will affect their whole situation, their whole family situation and their children. (respondent 6)*

#### Sensitive subject that can evoke difficult memories

In addition, some respondents brought up the sensitivity of some themes in the mental health material, such as trauma and reconciliation that could trigger difficult memories of war, hostilities, flight and losing family members. Talking about sensitive subjects in larger groups where people are not very familiar with each other such as civic orientation classes sometimes posed a challenge. This challenge was amplified when it came to the loss of a spouse or a child in a traumatic way.

*The most important thing is to give them room to speak about what they feel and try to listen to their stories. But the problem is that sometimes it’s sensitive stuff that might not be good to share in front of everybody. It might be something personal. But at the same time; if the person wants to talk in front of everybody there could be a point with that. It might lead to a bit of unease (in the classroom) but it’s not so dangerous and could motivate others to talk about their troubles. (respondent 5)*

A few respondents reflected on the fact that although they had received training, they were not specialists in mental health. For them, this meant that they were vigilant when bringing up subjects that could reminisce of traumatic events and prepared to follow up after class as well as refer to appropriate care or social services if needed. Some mentioned that in particular filmed material needed to be introduced extensively as it could trigger difficult memories.

#### Material and methods not adjusted for larger groups

A few respondents articulated that some of the material and methods, designed for the conversation circles (consisting of small groups) about mental health, needed adaptation and adjustment to be used in larger groups, i.e., CO classes. An example was the trauma theme related to the pre- and during- migration phases, where they perceived that the material was intended for smaller groups. However, the majority of the respondents expressed that they could overcome this barrier by being prepared and making certain adjustments ahead of discussions in larger classrooms, e.g., finding alternative exercises. Most respondents had used some or all elements of the course in their CO classes.

Another adaptation that was articulated by some was to adjust the material for those with no or low education. Adapting material to different educational backgrounds was something the respondents were used to within their work as civic communicators.

*I am using the trauma and self-care (material). There is some complicated information about the brain and specific terminology in Swedish. I read it over and over to be able to understand the framework and to be able to recount it in a simple way to participants who are low educated. […] I read it again and again, about reconciliation and acceptance. It’s really valuable. (respondent 1)*

#### Few arenas to talk about mental health during early post-migration

Some respondents reflected that there were few arenas to talk about mental health issues for newly settled migrants. Decreased funding due to dropping numbers of incoming refugees was also seen as problematic. As civic communicators, they interacted with and understood the need for mental health promotion in the group.

*Maybe politicians don’t understand and municipal authorities don’t understand, but we who have them (newly settled migrants) every day and communicate with them every day in civics classes see that there is a need (mental health), and if I can use my knowledge in the future that would be good. (respondent 3)*

Civic orientation classes were described by some respondents to be suitable for addressing mental health issues as they take place in the early phase of resettlement. As some explained, civic orientation and language teachers were the only ones who met newly settled migrants regularly over a long period. They further reflected that the civic orientation should start with mental health themes so that participants could benefit more from other themes in the civic orientation as well as other introduction activities.

*This (mental health information) should be given at the start of every civic orientation class. So before one talks about what applies here and how things work in Sweden […] one should talk about mental well-being to each and everyone that starts in a new group, to start with this I believe. (respondent 2)*

The separate mental health conversation circles aimed at the newly settled migrants were seen as a good complement and opportunity for those with more needs. Some also commented that access to mental health information and counseling is uneven across the country and fails to reach some in need of it. A few respondents commented that more adults could be reached with mental health information through the schools and centers for mother tongue teaching.

### Becoming aware of the mental health journey

The third theme refers to the course content, e.g., newly gained knowledge and its application. The majority of the respondents reflected extensively on the course content. All of the respondents found the course content and material on mental health to be useful and relevant both for civic orientation classes where they had already used their newly gained knowledge, and for leading conversation circles (in the future). Although the course was perceived as concentrated and intensive by some, all of the material was seen as important. Further, several respondents reported that course content had helped them personally to understand and heal from certain traumatic events.

#### Acquired new theoretical knowledge

All respondents reported that they had acquired new theoretical knowledge about many aspects of mental health including differences between various symptoms (e.g., stress and trauma). All themes; trauma, stress, grief and reconciliation, and how children and youth react to difficult experiences were brought up and discussed by the respondents. They reflected on them as being important and having deepened their understanding of mental health.

*Something very interesting for me was that you become aware of the mental ill health journey. How it starts, and then how life, your whole life can be affected, and then what we can do as civic communicators or conversation circle leaders or someone who meets a person with mental ill health. How I can act in this journey or at the point we’re at as I see it. Does the person have enough on their plate or can I add another step? If I’m going to explain it: when you start to talk about your troubles, then you have taken the first step ahead to find relief. Our role is to push them to take the next step. Tell: you might find relief or at least take the next step in this journey. (respondent 5)*

Some respondents stated that having theoretical knowledge has given them the necessary tools to overcome potential barriers more effectively and talk about mental health during their classes. As a result of their theoretical understanding, they perceived that with proper guidance, talking about difficult events could be beneficial for those who wanted to voice them.

*[…] You put words to those (difficult) feelings that we’ve had and we can use it in a more professional way towards our participants. There is more substance to it, there is more understanding of why they feel as they feel, and there is nothing wrong with it. So it’s very important to say that it’s ok to feel insecure and unstable. There is a reason for that. (respondent 2)*

Although the theoretical material was deemed relevant and useful by all, a few respondents reflected that some topics were missing, such as mental health related to sexual orientation and transgenderism. They lacked the means to address it in classrooms when needed.

#### Better at identifying ill health and interacting with participants

The majority of the respondents felt that following the course, they had become better at *identifying* participants who suffered from various mental ill health symptoms such as stress and grief. They understood better how people think, act and react when they are unwell. Examples given as signs of mental distress included participants being silent and absentminded in classes, not engaging, and not being able to focus or understand the content.

*I’m more observant now because I know more about symptoms. Then it becomes easier to see what the person suffers from by looking at their appearance, their behavior, how active they are, how much they engage when they speak and what examples they give, how many examples they can give when we ask certain questions, and what their answers are. The effect…you can see how they react. You see it in their reactions. (respondent 8)*

Furthermore, the respondents perceived that they were better at *handling* and *interacting* with participants that displayed signs of stress and other symptoms as they now had the tools to do so. Ways described by several respondents to make participants comfortable to talk, (based on Motivational Interviewing) were to use open-ended questions, make eye contact, show interest and respect, not interrupt and employ non-judgmental listening techniques.

*Some people are unwell (in class) and you can see that they don’t want to open up. And that’s what I learned, how to act towards them, ask them and make them talk. And to listen very carefully to them and show interest. (respondent 1)*

A few respondents also added that they had learned to acknowledge and validate what these participants had been through and pause before going on to the next step. Another way inspired by the course to handle and interact with participants, was to do certain small energizing physical activities or relaxing breathing exercises within the classrooms to alleviate some stress.

*So you get tools and other things that you didn’t think about before. Like if someone is sad all the time or someone just cries suddenly. How to handle those situations. Why it happens and why someone does not open up in front of the group. (respondent 8)*

Some reflected that it was better to listen carefully and have a deep conversation where participants felt listened to and seen than rushing to go through the curriculum at hand. A few articulated that they had understood the importance of following up with those who shared difficult memories and experiences in larger classes. One way that was described was to meet them separately after class to give individualized advice and refer them to appropriate care or authority if needed. Many expressed that they better understood when to refer participants to care or competent authorities. Some even recounted that they called and made appointments for participants, especially when they were in distress or had difficult circumstances such as experiencing domestic violence. In terms of referrals, one respondent reflected that specific information on how to identify suicidal persons was lacking in the course.

#### Using new material and more practical elements in the classroom

As a result of taking the course, all of the respondents had started using the new material that they had gotten in the civic orientation. Two of the respondents had also used it in conversation circles. The films and practical exercises were seen as especially valuable as they could be used for smaller or larger groups and be adapted if needed. A few participants felt that more guidance would have been useful on how and when to use the films as well as specific information on what they were targeting as they could contain several themes. Others felt that with a brief introduction of the themes beforehand they could guide their participants as to what they were about to see. All respondents felt that they could use the exercises from the course in their classrooms later on. Many of the respondents saw the fact that the material and exercises were pre-tested (on them and others) as a major benefit.

*So I don’t have to google and find material and then test what material is the most appropriate for the group, I have the material ready and available[…]You feel safe when you meet the group and know what to do, step by step. (respondent 1)*

Above all, the films and exercises offered a good way to start conversations about difficult subjects. The films, some reflected, were helpful as they offered consolation that others were going through the same difficulties. They also gave hope and inspiration. Several respondents mentioned that the exercises about reconciliation and acceptance were useful because of their simplicity. In addition, the role-plays and other exercises were perceived as good for understanding group dynamics and how to handle challenging situations in the classrooms such as conflicts between participants.

Many of the respondents reported that the exercises that they had tried out in the course had been therapeutic for them and useful in their personal lives. A few recounted that they had used some exercises and techniques at home for instance to calm a stressed or angry child or on themselves.

*It helped me in my life as well. As a person I am also stressed, I’m an immigrant and I don’t have a family here so it (the course) helped me a bit to understand what I have in my head. (respondent 3)*

The written material was seen as a good to have to go back to. While all used the material, some respondents reflected that they adapted the material for larger classrooms. For instance, they added their own exercises and material to it, based on previous experiences of teaching newly settled migrants.

#### Inspired and more secure as leaders

As a result of taking the course, a majority of the respondents expressed that they had become clearer about and more secure in their roles as conversation circle leaders as well as leaders of larger classes. One reason was that they had their knowledge and praxis confirmed by the course. They were also inspired and motivated both by what they learned as well as how the teachers led the course. The teachers were perceived as well prepared, knowledgeable, and stringent as well as very respectful by all of the respondents. They were also appreciated for the way they led the practical exercises. By observing the teachers and using their techniques, some respondents remarked that they become more successful in leading their classes:

*I have noticed that they share more now than they did before. I think it all goes back to how we work as communicators. (respondent 2)*

The respondents who had started their conversation circles reported that it was important to have a proactive approach and establish trust during the first lessons. One respondent recounted that he shared with his participants his experience of mental ill health related to migration, to de-stigmatize the issue of mental health and illustrate the benefits of sharing. Overall, they perceived the conversation to be at the center, and their task to lead it (not necessarily having all the answers), which in turn helped them to set boundaries for their roles. Some explained that the most important thing was to motivate the participants to talk and to be a good listener.

*The most important thing is to motivate them to talk and be a good listener. And give them room to finish their stories and focus on what they say instead of conveying things that we have in store. It’s important to plan, but the most important thing is to try and give the participants room to express what they want*. *(respondent 5)*

With the structured material and exercises as well as inspiration from the course leaders, many respondents felt that they could lead with more confidence and create a more relaxed atmosphere. This applied also to the larger classrooms. Dealing with mental health was seen as requiring more professionalism than other themes in the civic orientation, for example, Swedish history, laws, and regulations. Several respondents also perceived that they had learned from each other’s experiences as communicators during the in-depth training course.

## Discussion

The study explored civic communicators’ perceptions of an in-depth training course on mental health, provided within the scope of a professional educational program for communicators. The findings suggest that the in-depth training course led to increased mental health literacy among respondents in terms of acquiring new theoretical knowledge about mental health, identifying mental ill health among participants, and using new methods to convey mental health information. The study also adds knowledge on mental health needs and barriers to talking about mental health among newly settled refugee migrants, as perceived by the civic communicators.

The respondents in the study expressed that the in-depth course had enabled them to attain new theoretical knowledge and new tools and methods to lead reflective conversations about mental health and well-being with participants in their CO classes and conversation circles. Further, they had benefited themselves from this knowledge and used it in their everyday life. These results are consistent with two components of the mental health literacy (MHL) concept ‘*Understanding how to obtain and maintain positive mental health* and *developing competencies to improve one’s mental health care and self-management capabilities*’ [45]. Based on the rich descriptions of how the respondents used the course material, tools, and methods to teach about topics varying from trauma, grief, and stress to reconciliation it can be hypothesized that the MHL of the participants in the CO classes and conversation circles was also increased. This hypothesis is also in line with the initial results from a pilot study on participants’ views on receiving in-depth mental health information [51], but further research is needed. Several studies have found that improving mental health literacy can be beneficial for the prevention of mental ill health [13, 52] even though evaluations of specific programs are often lacking [13, 45].

The respondents expressed that they had become better at identifying ill health and interacting with participants with perceived mental problems. This too is in line with increased MHL [45, 46]. However, they also stated that while some individual-level needs (e.g., needs related to stress management, coping strategies and milder trauma) could be addressed with mental health promotion and increased mental health literacy [45], other needs require health care and assistance from social services. In this aspect, the in-depth course provided health communicators with insights not only as to *who* might be suffering from mental ill health but also to what degree self-management was applicable, i.e., *when* it was appropriate to refer to health care.

In addition, the respondents reported that they had become more confident to teach and resolve challenging situations in classrooms. Previous research suggests that intention, confidence, and feeling capable of aiding others are strong predictors of the quality of health related helping behaviors [14, 53]. Furthermore, the respondents reported having benefited themselves from the knowledge, and applied some of the practical exercises in their day-to-day life. This is in line with having increased MHL self-management capabilities and research on benefits of being a peer helper on one’s own well-being [54].

Some of the course material was seen as more suitable for smaller groups, e.g., conversation circles, and needed adaptation for use in larger groups. In terms of what was lacking in the course, two areas were identified; information on how to identify suicidal persons and information on issues related to sexual health and rights. Both topics should be considered in future in-depth courses.

The respondents expressed that mental health needs were widespread among newly settled refugee migrants in their classes and intertwined, i.e., connected to both pre-, during-, as well as post-migration stressors. This is in line with the vast literature on mental health needs and factors affecting health and integration in the post-migration phase [2, 55, 56]. The information needs related to mental health were perceived to be adequately covered during the in-depth course, however some challenges and ill health related to work and integration issues were also discussed from a structural point of view, i.e., needing attention from decision-makers. These results are in line with studies that have explored the health and mental health needs of refugee migrants stressing the importance of the resettlement context [57] and migrants’ life situations [32, 58].

The identified barriers were described as multi-layered, i.e., related to both the individual and the structural levels. At individual and group levels, barriers to talking about mental health, such as stigma and the sensitivity of the subject, was addressed and respondents reported that they had acquired new tools to overcome some of the barriers. Practical exercises and films were mentioned as especially helpful. This is in accordance with the component of MHL ‘*decreasing stigma related to mental disorders*. Stigma is a well-known barrier to increasing MHL [29, 30]. Studies on stigma and mental health include perceptions of stigma in different migrant populations, and its effects on accessing mental health services [31, 32, 59]. In our study, the respondents perceived that conveying the material in their languages and having experienced migration as refugees themselves helped them to gain the participants’ trust, making it easier to guide participants to available resources, i.e., enhancing their help-seeking efficacy. This is in line with research on the positive effects of cultural mediation in decreasing language and cultural barriers and facilitating communication and understanding [60]. Structural barriers such as lack of opportunities or arenas to promote mental health during the establishment phase were also mentioned. The CO was mentioned as an arena where mental health promotion and MHL, could be increased to strengthen competencies and self-management capabilities in newly settled migrant populations. This was seen as something that would increase participants’ well-being and learning from language courses and other introduction activities in the establishment phase, thus promoting integration. The potential of migrant integration policies to include health promotion for better health and integration outcomes have been highlighted in other studies [8, 41].

### Methodological considerations

A limitation of the study was that the interviews were conducted in Swedish. Although all respondents were proficient in Swedish (to different degrees), it was not their native language. They might therefore have been limited to express themselves fully in the interviews. Another possible limitation was the uneven distribution of men and women among the respondents. However, it was not evident from the data that there were differences in views based on the respondents’ language proficiency or gender. All individuals who were eligible for inclusion in the study consented to participate, which meant having maximum diversity in terms of perceptions about the course, both positive and negative. We see the risk of respondents having felt obliged to participate in the interviews as minimal, as there was no apparent advantage in doing so. Further, having been trained and working within the framework of MILSA, with continuous evaluations of projects, the respondents had an understanding that any “gain” in terms of implemented changes to the course would be protracted. Nevertheless, it cannot be entirely ruled out that they felt obliged to participate.

A strength of the study was that three authors, with different educational and ethnic backgrounds, coded the transcripts, which increased the reliability and validity of the interpretation of the data. The fact that the interviewing researcher had previously been a lecturer for the respondents could mean that they felt at ease and could share negative views. At the same time, they might have been inclined to report more positive than negative views. However, the results include negative views as well as suggestions for future courses. As for possible biases introduced by the interviewer’s Swedish background, we believe that they were countered by discussing the results in a triangulation process involving authors (JW, MA, EÅ) with different backgrounds and experiences.

As for the transferability of findings, we believe that the results could be applied to similar settings and similar populations. This is especially true for the observed mental health needs and barriers to talking about mental health. As for the results on knowledge application, transferability is contingent on similar educational components.

## Conclusions

The findings suggest that the in-depth course increased MHL competence among respondents in the study. The mental health in-depth training course led to the attainment of new knowledge and new tools that enabled the civic communicators to lead reflective conversations about mental health and well-being with newly settled refugee migrants. Mental health needs were seen as intertwined, i.e., related to both pre- and post-migration experiences. Barriers to talking about mental health were multi-layered and included stigma and lack of arenas to promote the mental health of refugee migrants. Increasing knowledge and mental health literacy among communicators and workshop leaders can facilitate the promotion of mental self-help capacities and resilience among newly settled refugee migrants, which in turn can support their resettlement process and integration.

### Implications

- In-depth courses in mental health for civic communications given in conjunction with the educational program for civic- and health communication, can increase communicators’ knowledge and enhance the delivery of mental health promotion targeted at newly settled refugee migrants.
- Some adaptation of the in-depth course material and tools is needed for use in larger groups. Information on how to identify suicidal persons and information on topics related to sexual health and rights should be considered in future courses.
- Civic Orientation can be a suitable platform to promote mental health among newly settled refugee migrants.

## Supplementary Information

**Additional file 1: **Short description of main themes in the *Ways forward after the flight material*.

**Additional file 2. **Interview guide.

## Data Availability

The analyzed data of the current study are available from the corresponding author upon reasonable request. To safeguard the identities of participants, the full interview data of the study, i.e., transcripts and audio files will not be made available to the public.

## Abbreviations

WHO: World Health Organization
CO: Civic Orientation
MILSA: Support Platform for Migration and Health
MHL: Mental Health Literacy
