# Supplementary material for: ”Putting words to their feelings”– civic communicators’ perceptions and experiences of an in-depth course on mental health for newly settled refugee migrants in Sweden
Source: BMC Health Serv Res. 2023 May 19;23:510. doi: 10.1186/s12913-023-09524-2 (PMC10198591; doi:10.1186/s12913-023-09524-2)
Supplement: Supplementary file 1 — Additional file 1: Short description of main themes in the Ways forward after the flight material. [file 12913_2023_9524_MOESM1_ESM.pdf]

Additional file 1. Short description of main themes in the *Ways forward after the flight* material

Health and well-being
Introductory theme where the group leader and participants reflect on health and well-being. The four dimensions of health is presented; physical health, mental health, social health and existential health. How to promote and strengthen one's health is discussed as well as how health and well-being can be expressed and viewed in different cultures.

Loss and identity
Participants are guided to reflect on their identity and relationships. They are inspired to see the significance of their connectedness with others and to see their lives as a whole. The idea is to recognize that their present lives and life before the flight are not separate parts, but will one day form a whole. It is stressed that life before the flight should not be forgotten or viewed as just in the past, but rather as an important part of the present. Films and exercises that facilitates talking about pre-migration experiences, experiences of loss and life in Sweden are included in this segment.

Grief and forgiveness
The objective with these themes is for the participants to gain an increased understanding of forgiveness and grief and to progress in their own processes of forgiveness and grief. The participants are given theoretical background and terminology related to grief, physical and psychological manifestations of grief, grieving and reconciling in a migration perspective. Mourning, forgiveness, responsibility/power and letting go is discussed and reflected upon through exercises and film clips.

Reconciliation and acceptance
The objective with the theme is for the participants to see an opportunity to accept and reconcile with what they cannot change. The objective is also to highlight capacities and build resilience. Terms such as guilt, shame and self-esteem is discussed. Part of the work of reconciliation is to have a conversation around what is painful, what is lost, how a different identity feels and to try navigating towards a new reality. Exercises and films are used as tools (see below).

Stress and stress management
Stress and migration related stress is discussed in this theme. Types of stress, e.g. short-term and long term/ongoing stress and good/bad stress is described. How stress can manifest physically and psychologically (pain, dizziness, sleep problems, concentration difficulties, irritability etc), is explained as well as short and long-term effects on health and relationships. Exercises, techniques and strategies on how to reduce stress are taught and some exercises are tried out.

Trauma and trauma conscious care

Participants gain an increased understanding of what traumatic stress is and how traumatic stress can manifest and affect the body and mind and life as whole. How trauma can be triggered in everyday life is also discussed as well as more severe trauma, i.e., post-traumatic stress (PTSD). Participants learn that it is normal to have a stress reaction after difficult and traumatic experiences and receive tips on what they can do themselves and when to seek help. Participants can share memories about difficult events, but it is stressed that they do not have to do that. Guidance on how to talk about individual's experiences is given as well as instructions to follow in situations where difficult emotions are triggered. Children's trauma and trauma conscious care is also discussed within this theme through material produced by Save the Children. Trauma conscious care focus on children's trauma and its different manifestations in terms of health and behavior. The link between children's trauma and adults' trauma is also discussed.

Conversation circles methodology

This theme consist of several parts including, conversation circle methodology, pedagogical methods and what the role of a conversation circle leader entails. It include training on how to plan, set up and lead a conversation circle meeting, how security can be created in groups and how different challenging situations that may arise in groups can be handled. The concept of empathy is discussed and how they as conversation circle leaders can relate to participants with possible trauma and mental ill health. As part of the pedagogical methods, examples of practical exercises (i.e., relaxing and energizing exercises) are given, as well as suggestions on how short films and exercises can be used to promote conversations about mental health related issues.

The film material is used in several themes and consist of three short films, focusing on identity, stress and traumatic stress respectively. The purpose of the films is to support conversations about migration-related mental ill health. They are based on interviews with people with own migration experience who have established themselves in Sweden, and with experts in migration related stress and mental health. The idea is to normalize feelings and reactions migrants may have in the early post-migration phase and confirm that they are not alone in these experiences. Further, the films provide suggestions on how to make everyday life manageable and increase a sense of hope for the future.
