# Supplementary material for: ”Putting words to their feelings”– civic communicators’ perceptions and experiences of an in-depth course on mental health for newly settled refugee migrants in Sweden
Source: BMC Health Serv Res. 2023 May 19;23:510. doi: 10.1186/s12913-023-09524-2 (PMC10198591; doi:10.1186/s12913-023-09524-2)
Supplement: Supplementary file 2 — Additional file 2. Interview guide. [file 12913_2023_9524_MOESM2_ESM.pdf]

Additional file 2. Interview guide

Introductory questions

Questions about age, sex, level of education, occupation/profession, country of birth, number of years with a residence permit in Sweden, number of years as a civic- and health communicator and in which language the respondent leads civic orientation classes.

General questions about perceptions and experiences of the in-depth course

- How did you feel participating in the course?
- What new knowledge did you gain by participating in the course?
- What do you think about what you learned in the course?

Probing questions on perceptions and experiences of the in-depth course

- How would you say that participation in the course affected the way you think about mental health?
- How would you say that participation in the course affected how you view your role as a communicator in the Civic Orientation when it comes to mental health?
- How would you say that participation in the course affected the way you work as a communicator when it comes to mental health?
- How would you say that participation in the course affected your ability to access, appraise and apply information about mental health?
- How would you say that participation in the course has affected how you collaborate with other people, associations and stakeholders to promote mental health of newly settled migrants?
- How would you say that participation in the course affected your ability to guide newly settled migrants to activities that other stakeholders offer to promote their mental health?
- What from the course, do you think you will benefit most in your work with new settled migrants?
- To what extent has the course made you ready to lead your own study circles about mental health with newly settled migrants?

Closing questions

- What do you think of the material you were given during the course?
- How did you experience the methods and practical exercises that you had to do or learn during the course?
- What was the best part of the course?
- Something you missed in the course?
- How could the course be improved?
- Anything else you want to share?
